# In Vivo Fat Quantification: Monitoring Effects of a 6-Week Non-Energy-Restricted Ketogenic Diet in Healthy Adults Using MRI, ADP and BIA

**DOI:** 10.3390/nu12010244

**Published:** 2020-01-17

**Authors:** Martin Buechert, Thomas Lange, Peter Deibert, Paul Urbain

**Affiliations:** 1 Department of Radiology, Medical Physics, Medical Center—University of Freiburg, Faculty of Medicine, University of Freiburg, 79106 Freiburg, Germany; Thomas.Lange@uniklinik-freiburg.de; 2 Institute for Exercise—und Occupational Medicine, Center for Medicine, Medical Center—University of Freiburg, Faculty of Medicine, University of Freiburg, 79106 Freiburg, Germany; Peter.Deibert@uniklinik-freiburg.de; 3 Department of Medicine I, Section of Clinical Nutrition and Dietetics, Medical Center—University of Freiburg, Faculty of Medicine, University of Freiburg, 79106 Freiburg, Germany; Paul.Urbain@uniklinik-freiburg.de

**Keywords:** ketogenic diet, low carbohydrate, non-energy-restricted diet, body composition, magnetic resonance imaging, magnetic resonance spectroscopy, dixon MRI, liver MRS, ADP, BIA

## Abstract

The ketogenic diet (KD) is a very low-carbohydrate, high-fat, and adequate-protein diet that induces many metabolic adaptations when calorie intake is not limited. Its therapeutic use in a range of diseases including cancer is currently being investigated. Our objective was to firstly assess the impact of a 6-week non-energy-restricted KD on the abdominal fat distribution and the hepatic fat composition in healthy adults. Body fat distribution and composition were measured by comparing magnetic resonance imaging (MRI) and spectroscopy (MRS) results with air displacement plethysmography (ADP) and bioelectrical impedance analysis (BIA) measurements. A total of 12 subjects from the KetoPerformance study were recruited for this ancillary study. Body mass index (BMI), total mass, total fat mass, total subcutaneous mass, and subcutaneous fat mass decreased significantly. None of the MRS parameters showed a significant change during the study. Even though the average change in body weight was >2kg, no significant changes in intrahepatic lipid (IHL) content could be observed. Total fat mass and total fat-free mass derived from MRI has a strong correlation with the corresponding values derived from BIA and ADP data. BMI and the absolute fat parameter of all three modalities decreased, but there were no or only minor changes regarding the fat-free parameter. Magnetic resonance imaging provides body composition information on abdominal fat distribution changes during a ketogenic diet. This information is complementary to anthropomorphic and laboratory measures and is more detailed than the information provided by ADP and BIA measures. It was shown that there was no significant change in internal fat distribution, but there was a decrease in subcutaneous fat.

## 1. Introduction

The ketogenic diet (KD) is a very low-carbohydrate (<10% of energy), high-fat (>60% of energy), and adequate-protein diet that without limiting calories induces a metabolic condition called “physiological ketosis” with increased levels of circulating ketone bodies [1]. The clinical application of KD includes its role as long-time proven therapy for intractable childhood epilepsy [2]. Its therapeutic use in a range of diseases such as type 2 diabetes, polycystic ovary syndrome, neurodegenerative diseases, and cancer is currently being investigated [3]. An energy restricted KD have recently become quite popular as a weight-loss diet [4], which is based on the carbohydrate–insulin model of obesity and the prediction of a greater rate of body fat loss during KD [5].

One aim of the *KetoPerformance* study [6], the main study of this sub-study, was to investigate whether KD as a fasting supportive diet could reduce fasting-related discomfort and increases the compliance with fasting strategies without compromising exercise capacity in cancer patients.

A review of the existing literature of KD’s effects showed that the available data is very limited. Only five small studies meeting our search criteria were identified [5,7,8,9,10], where three included performance athletes [7,8,9], one involved a high protein diet, which was probably not ketogenic [8], and one examined overweight and obese men [5]. The objective of the main study therefore was to firstly assess in a larger trial the impact of a non-energy-restricted 6-week KD in healthy adults beyond cohorts of performance athletes on physical performance (endurance capacity and muscle strength), body composition, and a range of blood parameters. Due to the specific composition of a KD, it induces many metabolic adaptations, e.g., increased levels of circulating ketone bodies and a shift to lipid metabolism. The hypothesis was that such metabolic adaptions may also affect the lipid distribution and lipid composition within the body and more specifically within the abdomen.

To access intra-abdominal adipose tissue measures, a variety of imaging modalities including computed tomography (CT), dual energy absorptiometry (DXA), magnetic resonance imaging (MRI) and ultrasound is used [11]. While CT is an accurate and relatively rapid measurement, it goes along with radiation exposure, sometimes limiting its use to single slice instead of volumetric acquisition. Radiation exposure is also a limiting factor for DXA, which is why in some countries like Germany this method is not allowed in children, young adults, and pregnant women [12]. The use of ultrasound imaging is limited to estimate subcutaneous fat thickness, while adipose tissue volume estimation is imprecise [13]. Air displacement plethysmography (ADP) is a densitometric method used to measure human body composition [14]. ADP is a fast and noninvasive measurement modality. However, in contrast to imaging methods, it only delivers overall instead of spatially resolved body composition values. Bioelectrical impedance analysis (BIA) involves a body composition assessment using electrical impedance measurements to estimate primary total body water, which then can be used to estimate fat-free body mass and, through the difference with body weight, body fat. BIA is considered to be acceptable for determination of body composition of groups and for monitoring changes in body composition within individuals over time. Single BIA measurements in individual patients, however, are considered to be subject to error [15] and as with ADP, it does not deliver spatially resolved values.

Recently, more sophisticated MRI methods have been developed to measure tissue distribution and composition, including spatially resolved lipid volumetry. Furthermore, local tissue composition including fat composition has been investigated with magnetic resonance spectroscopy (MRS). However, MR examinations come at the expense of higher costs compared to other methods. In this study, we used MRI and MRS to measure the effect of a 6-week ketogenic diet on the abdominal fat distribution and the hepatic fat composition. This is the first study in humans in order to quantify the effect of a ketogenic diet on liver fat content and composition. Results are compared and related to accompanying measures of the main study, including anthropometric and laboratory data as well as BIA and ADP results. One focus lies on the comparison of the spatially resolved MRI results with the overall values from ADP and BIA investigations.

## 2. Materials and Methods

### 2.1. Participants

Seventy-two volunteers were recruited for the *KetoPerformance* study [6] (registered at germanctr.de as DRKS00009605), out of which 42 could be allocated to the KD intervention and successfully finished the 6-week intervention study. Twelve of these 42 subjects were recruited for the present ancillary MR study. The subjects’ age was in the range of 30–67 years (mean 48.4 ± 11.3) and the BMI range was 22.3–33.3 kg/m^2^ (mean 25.8 ± 2.8). In addition to the inclusion and exclusion criteria of the main study [6], MRI contraindications including unsafe ferromagnetic or functional MR implants as well as claustrophobia applied. All subjects gave their informed consent for inclusion before they participated in the study. The study was conducted in accordance with the Declaration of Helsinki, and the protocol was approved by the Ethics Committee of the Albert-Ludwig University Freiburg (494/14).

### 2.2. Study Design and Intervention

This study had a single arm before-and-after comparison design. The experimental intervention consisted of a KD without caloric restriction lasting six weeks (42 days) with a preceding preparation period including detailed instructions during teaching classes and individual counselling by a dietitian. Day one and day 42 will subsequently be denoted as PRE and POST, respectively. More details about study design and intervention as subjects of the main study are given in reference [6]. 

### 2.3. Data Acquisition

Except of MR-examinations all testing procedures were performed at the Institute for Exercise- and Occupational Medicine in the morning between 07:00 and 09:30 after an overnight fast lasting at least 8 h. The subjects were not allowed to exercise the day before, and were advised to arrive to the examinations without any physical effort. Our endpoints are hereafter described in the chronological order recorded at PRE and POST. Fat mass (FM) and fat-free mass (FFM) were determined via air displacement plethysmography (ADP) using the BodPod device (Cosmed USA Inc., California, CA, USA), which was calibrated prior to each use according to the manufacturer’s guidelines. By using bioelectrical impedance analysis BIA 2000-M (Data Input, Pöcking, Germany) following a standardized procedure according to guidelines [16], body compartments FM, FFM, and body cell mass were determined. More details about ADP and BIA methodologies of the main study are given in reference [6]. 

All MR experiments were performed on a 3T MR system (Magnetom Trio, Siemens Healthineers, Erlangen, Germany) at the Department of Radiology, Medical Physics. Two six-channel body array coils, a spine array coil integrated into the patient table for signal reception, and a MR protocol employed in previous studies [17,18] were used. The subjects underwent the following MR protocol on the first day of the study, before KD intervention (PRE) and the last day of the study, which was day 42 (POST). A Dixon-based sequence [19] covering the abdomen at least between the top of the femoral heads and the liver apex was used for fat/water imaging.

**Figure 1:**
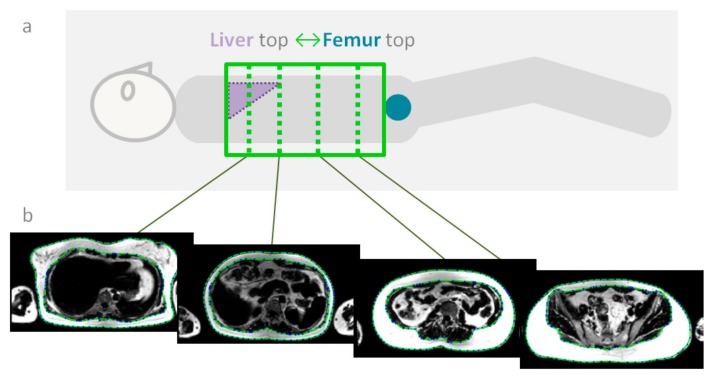
(**a**) Symbolic view of the definition of the abdominal magnetic resonance imaging (MRI) imaging volume (green box) between the top of the femoral heads (blue circle) and the liver (purple triangle) apex. (**b**) Corresponding MRI slices at four in (**a**) marked exemplary positions within the imaging volume. The dashed green lines within the MR-images show the segmentation borders of subcutaneous fat volume. Reconstructed images are based on the fat-water Dixon MR-images acquired with a spoiled gradient echo sequence (repetition time TR = 171 ms, four echo times TE = {1.11, 2.89, 4.67, 6.45} ms, a slice thickness of 6 mm, and an in-plane resolution of 2.3 mm).

Figure 1 gives an overview of the measurement locations within the abdomen. Furthermore, under free breathing and prospective acquisition correction (PACE) based on navigator triggering, liver MRS was performed using single voxel point-resolved spectroscopy (PRESS). The measurement voxel (3 × 3 × 3) cm^3^ was positioned in the lateral part of the liver, avoiding the liver edges as well as contamination from larger blood vessels. Using this setup, non-water-suppressed as well as water-suppressed MRS data (Figure 4) with 64 spectral averages were acquired using an echo time (TE) 35 ms and a minimal repetition time (TR) of 1 s.

### 2.4. Data Post Processing

Fat and water images were reconstructed from the acquired multi-TE gradient echo MR data using the graph cuts algorithm [20], and intra-abdominal and subcutaneous fat volumes were determined. Using mean density values [21,22] allows estimation of corresponding fat and fat-free masses [17]. For tissue segmentation and fat quantification, a MATLAB (The Mathworks, Inc., Natick, MA, USA) based analysis pipeline was used [17]. This included a semi-automatic segmentation to quantify intra-abdominal fat, mainly visceral adipose tissue (VAT), and abdominal subcutaneous adipose tissue (SAT) and their corresponding fat-free tissue shares separately.

The liver MR spectra were fitted and quantified with LCModel [23], using a dedicated analysis protocol for lipid detection in the liver. The lipid signal was modeled with peaks at [0.9, 1.3, 1.6, 2.1, 2.3, 2.8, 4.1, 4.3, 5.2, 5.3] ppm by LCModel. Quantification results determined with Cramer-Rao lower bounds larger than 20% were rejected for further analysis. For determination of the intrahepatic lipid (IHL) content, the fat signal (FS) was quantified as the sum of the integrated lipid peaks at 0.9, 1.3, and 1.6 ppm, while for the water signal (WS), the integrated water peak in the non-water-suppressed spectra was taken. The integrated signals were corrected with the relaxation constants reported by [24] and the IHL content was calculated as IHL = FS/(FS + WS). From the quantified lipid resonances, mean chain length, saturated lipid component, total unsaturated lipid component, and fraction of unsaturated lipids were determined as described by reference [25].

Data acquisition of fasting blood parameters as well as fat mass (FM) and fat-free mass (FFM) determined via air displacement plethysmography (ADP) and bioelectrical impedance analysis (BIA) were part of the main study and are described elsewhere [6]. 

### 2.5. Statistics

Bland-Altman-diagrams were created to evaluate the agreement among BIA and ADP results and to allow identification of any systematic difference between baseline and follow up measurements of the same modality. A Wilcoxon signed-rank test was used to check for distribution differences in the matched samples of PRE and POST values and asymptotic significance values (2-tailed) were calculated. Pearson correlation coefficients were calculated for investigating associations between variables. The data were analyzed using IBM SPSS 24 for statistical analysis (IBM, New York, NY, USA).

## 3. Results

### 3.1. MRI

All twelve subjects finished PRE and POST MR examinations a well as the corresponding ADP and BIA assessments. Supplementary Figure S1 displays (b) Dixon fat/water MRI as well as (c) non-water-suppressed MRS example data and the corresponding (a) slices and voxel positioning, respectively. Data of one patient had to be excluded since the patient could not follow breathing commands during the POST examination and accordingly the measured MR data were corrupted. For all subjects, fasting blood parameters as well as fat mass (FM) and fat-free mass (FFM) data, determined via ADP and BIA within the main study, were available.

For total body weight normalized values of BMI and results derived from MRI data of total subcutaneous mass, subcutaneous fat-free mass, and subcutaneous fat mass changed significantly from PRE to POST, as listed in the lower part of Table 1. This decrease went along with a decrease in body mass index for all subjects (Supplementary Figure S2). All values calculated for the total abdominal masses and internal masses did not show significant changes.

**Figure 2:**
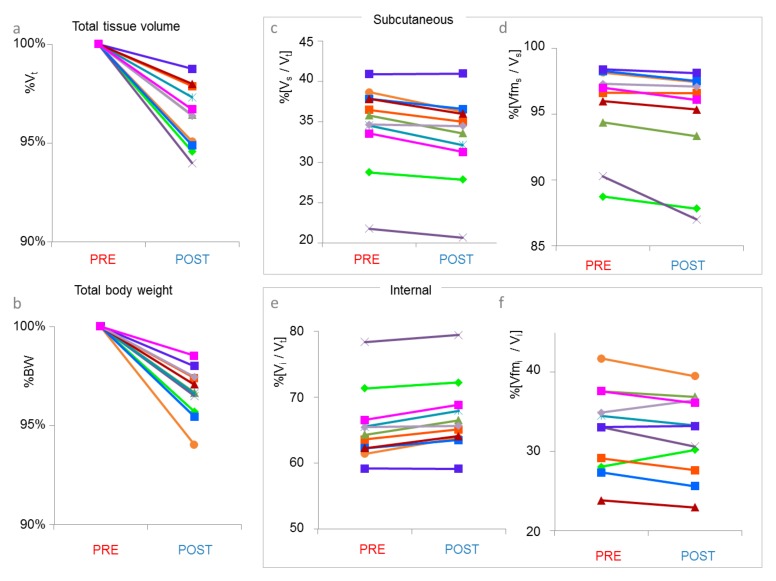
Line plots showing changes of individual subjects’ MRI data. Each plot on the left contains PRE values before diet and on the right contains POST values after six weeks. (**a**) Total tissue volume V_t_ normalized to the total tissue volume PRE. (**b**) Total body weight normalized to the total body weight PRE (**c**) Subcutaneous volume V_s_ normalized to V_t_. (**d**) Subcutaneous fat mass volume Vfm_s_ normalized to V_s._ (**e**) Internal volume V_i_ normalized to V_t_. (**f**) Internal fat mass volume Vfm_i_ normalized to V_i_.

An intra-subject comparison of PRE vs. POST MRI results showed a systematic decrease in total abdominal volume between the top of the femoral heads and the liver apex for each subject prior to diet and six weeks after diet initiation (Figure 2a), which accompanied an decrease in body weight for all subjects (Figure 2b). Volume ratios of fat-free mass/total mass and fat mass/sub volume mass for the subcutaneous and the internal sub volumes are plotted in Figure 2c,d and Figure 2e,f, respectively. None of the values reached given significance levels for the differences of PRE and POST values. Total volume and the ratios of subcutaneous volume/total volume, subcutaneous fat mass volume/subcutaneous volume decreased between pre and post for all subjects. Correspondingly the ratios internal volume/total volume and subcutaneous fat-free mass volume/subcutaneous volume increased between pre and post for all subjects. 

### 3.2. Modality Comparison

**Figure 3:**
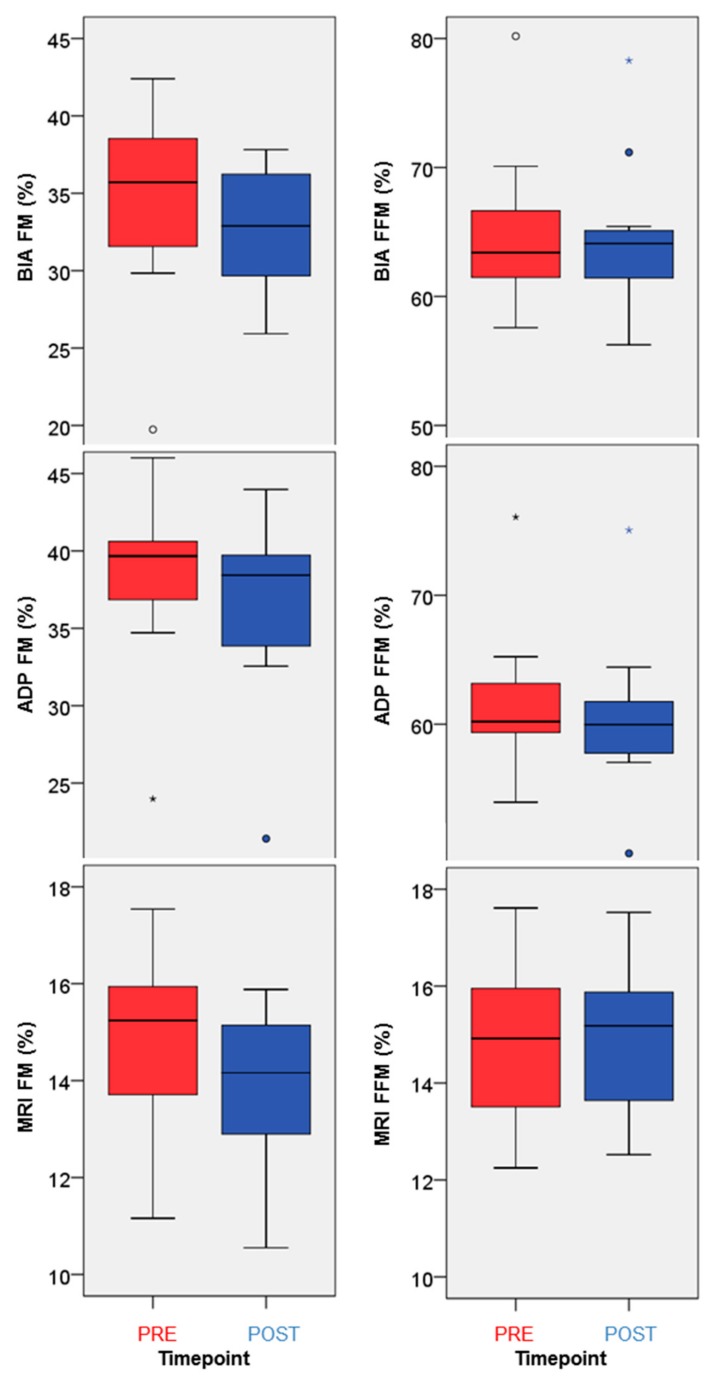
Box plots fat mass (**right**) and fat-free mass (**left**) as percentage of the body weight measured by BIA (**top**), ADP (**center**) and MRI (**bottom**). X-axis labels before diet are ‘PRE’ and after six weeks are ‘POST’ values. MRI data restricted to the imaging volume, BIA and ADP values for the whole body. Extreme values are represented by circles (‘out’) and asterisks (‘far out’). Abbreviations: FFM = fat-free mass, FM = fat mass.

For comparison, Figure 3 displays changes of fat mass (left) and fat-free mass (right) measured by air displacement plethysmography (ADP), bioelectrical impedance analysis (BIA), and magnetic resonance imaging (MRI) results. Supplementary Figure S3 shows each modality Bland-Altman-Plot comparing PRE and POST examinations. This view on an individual subject level reveals a systematic and consistent decrease of fat mass in all subjects except for one. Fat-free mass showed the same direction of changes in individual data for ADP and MRI results. BIA fat-free mass results did not reveal a consistent development over all subjects. On a group level, BMI and fat parameters of all three modalities decreased significantly, but there was no such uniform change regarding the fat-free parameter (Table 1).

Bland-Altman plots shown in Supplementary Figure S4 compare ADP and BIA results for fat and fat-free mass. Through statistical analysis, it was found that total fat mass derived from MRI has a strong correlation (*p* < 0.001), with the corresponding values derived from BIA data with a correlation coefficient of 0.815 and from ADP data with a correlation coefficient of 0.834, respectively. The same was found for total fat-free mass with correlation coefficients of 0.906 for MRI vs. ADP and 0.904 for MRI vs. BIA. Correlations coefficients between BIA and ADP data were 0.937 for fat mass and 0.982 for fat-free mass with *p* < 0.001.

### 3.3. MRS

**Figure 4:**
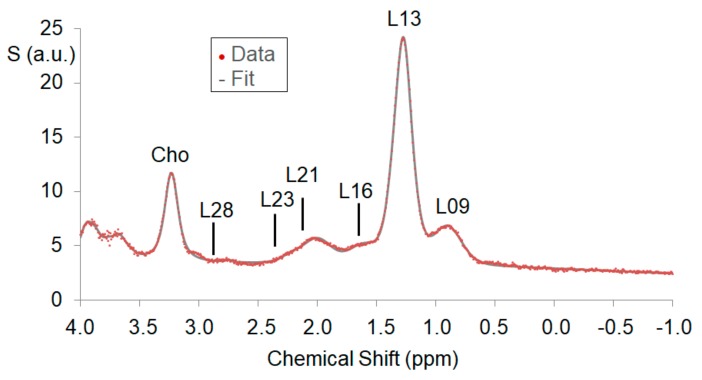
Example water suppressed liver spectra with main peaks marked: a methylene (CH2) peak at 1.3 ppm, a methyl (CH3) peak at 0.9 ppm, α-olefinic and α-carboxyl peaks at 2.1 ppm, a diacyl peak at 2.8 ppm, and a choline peak at 3.2 ppm. The model fit (thin underlying grey line) of the single components fits the measured data (red dots) very well.

Figure 4 shows example liver spectra with the quantifiable lipid peaks. One subject had to be excluded from the MRS analysis due to erroneous voxel positioning. The LCModel analysis could be performed for all other subjects. The IHL content as well as saturated lipid component and fraction of unsaturated lipids could be determined for all spectra. The mean chain length parameter could only be quantified without accounting for the resonances L52 and L53, which could not be quantified with sufficient accuracy (Cramér–Rao lower bound CRLBs < 20%) in several subjects. The total unsaturated lipid component could not be determined in all subjects and was therefore excluded from further analysis. 

**Figure 5:**
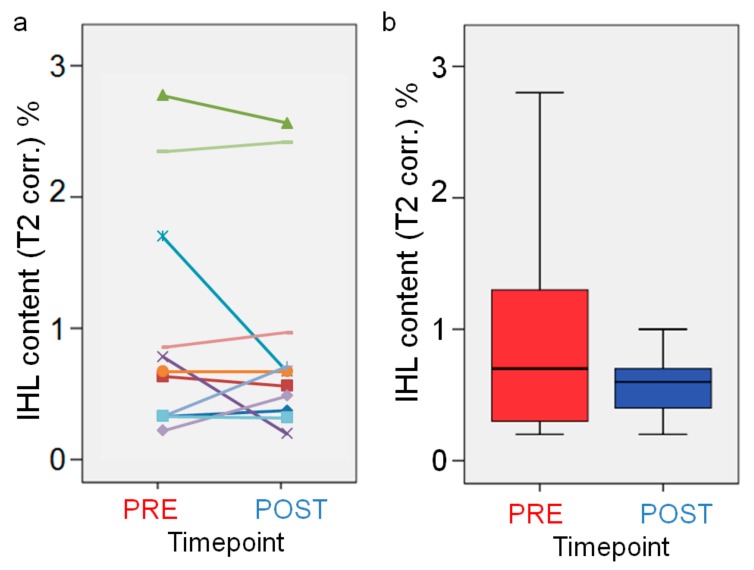
Mean intrahepatic lipid (IHL) values for PRE and POST measurements. Scatter plot of individual values (**a**) and box plots of grouped values (**b**).

None of the MRS parameters showed a significant difference between PRE and POST examination. Figure 5 shows the derived mean intrahepatic lipid (IHL) values for PRE and POST measurements. Changes of IHL between PRE and POST examination were heterogeneous between the subjects and on a very low level were mostly below 1%, as seen in Figure 5a.

## 4. Discussion

The primary purpose of this before-and-after comparison study was to evaluate the effects of a proven non-energy-restricted KD on body composition parameter and intrahepatic lipid content. After the 1-week transition phase, urinary ketosis was detectable on 97% (69%–100%) of the days, revealing a very good compliance to the KD [6]. While low-technology measures such as BMI, waist circumference, and waist-to-hip ratios can give some indication on the locations of fat deposits in the body, they provide little knowledge about local body composition, since they are indirect measurements. There is also uncertainty about how these measures perform across diverse ethnic groups since many earlier studies are based chiefly on Caucasian populations, and hence, it remains unclear whether derived relationships are consistent in non-Caucasian populations [26]. 

Magnetic resonance methods like MR-fat-water-imaging and liver spectroscopy, developed or optimized for this purpose in the context of obesity research, were employed to access such parameters on an individual level within this study. With full volume coverage and spatial resolution in the mm^3^-range, MRI is the gold standard for in vivo body composition measurements [27]. In addition, air displacement plethysmography and bioelectrical impedance analysis data available from the main study were compared to the MR findings. We found a strong correlation of fat mass as well as of fat-free mass derived from ADP, BIA, and MRI, even though the basic measurement principles are quite different and there are differences in the body coverage of the methods.

BIA estimates total body water indirectly via electrical impedance measurements [28]. The subtraction from body weight leads to an estimate of body fat mass. ADP measures body volume, allowing the estimation of fat and fat-free mass in combination with body weight using empirically derived equations [29]. MRI directly measures the volume and type of body tissue, allowing direct calculation of body composition fractions by segmenting the data [30]. MRI data in this study was limited to parts of the trunk, as shown in Figure 1. Even though there are individual variations in mass distribution over various body parts, the individual mass changes from PRE to POST measured by ADP, BIA, and by MRI showed the same trend. Variations may be attributed to the fact that according to body segmentation data, the whole trunk accounts for 43% of the body mass [31], which leads to approximately 20%–25% of the whole body mass accounting for the MRI-examined part in this study. Changes in the remaining part of the body go unnoticed by such an abdominal MRI protocol. However it is known that with weight loss the waist/hip ratio does not change, but the waist/thigh ratio decreases [32], since there is a relatively greater deposition of fat at the waist than on the thighs. This might be an indication that the good correlation values may be read as if changes in the rest of the body are at least not opposite to the changes in the trunk. Correlation values r < 1 can be interpreted to indicate that changes are smaller than in the trunk. MRI measurements with restricted body coverage like the used abdominal MRI-protocol therefore seem to be a good estimation of the overall changes in the body. Alternatively a whole-body MRI protocol would have to be applied [17]. This would allow exact measurements of spatially resolved fat distribution in the whole body [33]. The disadvantage is a significant longer acquisition time. Since this usually also requires covering not only the abdomen, but the whole body with surface array receiver coils, patient comfort and therefor the overall patient acceptance rate might be reduced.

The good correspondence in the results of weight and body composition from ADP and BIA in this subgroup with the findings of the main study indicates that even though the sample size was limited, it seems to be representative of the larger group of the main study. Significant losses of fat mass and, albeit to a lower extent, of fat-free mass via BIA were found, while the significance levels of the losses in fat mass and in fat-free mass derived from ADP data were much higher. Except for one subject on an individual level, the Bland-Altman plots revealed a consistent decrease in fat mass independent of the used method. However, the absolute values differ, which is not surprising since the examination volume and therefore the absolute covered fat mass examined was different in MRI. The Bland-Altman plots also reveal that the changes for fat-free mass measured by BIA are incoherent; over all subjects they varied from small decrease to small increase over the subject group. This may be attributed to the known limitations of single BIA measurements in individual patients [15]. While BIA values are mainly influenced by body water within extremities, it is of limited use regarding the tissue composition within the abdomen. The latter can be very precisely measured by MRI [33], which is an additional benefit compared to BIA examinations.

MRI data is in agreement with these findings showing significant losses in fat mass but not in fat-free mass. In addition to this, the possibility of MRI to differentiate the localization of the tissue by segmentation into various compartments or even into muscles and organs allows a more detailed view [34]. In the analysis of this study, segmentation into the two compartments of subcutaneous and internal tissue was done. This reveals that the overall losses in fat-mass are mainly attributed to losses in the subcutaneous fat mass and only to a small extent to changes in internal fat mass. Changes in fat-free mass are not observable for internal tissue. This also shows that the overall losses in abdominal mass leading to reduced body weight is to a large extent a result of the losses in subcutaneous fat mass. 

The found mild weight loss over the entire 6-week KD period is consistent with non-energy-restricted KD studies, although one study suggested that body fat loss slowed on transition to KD [5,7,9,10,35] possibly because of augmented utilization of body protein. The seemingly discrepancy to the finding of unchanged mean energy intake is discussed in more detail in the main study [6]. The main study showed that a non-energy-restricted KD leads to a significant but mild weight loss over the entire 6-week KD period, although mean energy intake did not change [6,36]. Even if the diet was supposedly not energy restricted, no measurements of energy expenditure or strict dietary compliance was documented and a slight reduction in energy intake could be possible. The KD predominantly impaired the endurance capacity but not the performance in the sub maximum area so that activities of daily living and training in the aerobic zone would not be impaired. The ADP measurement, which is based on the same principles as the gold standard method of hydrostatic weighing [14], revealed that weight loss consisted in equal parts of reductions in fat and fat-free mass, which is consistent with the results of the present subgroup analysis. In this study, intrahepatic lipid content was measured by MRS. IHL values were lower than reported in other studies investigating healthy subjects [37,38], however the BMI of their subjects were higher compared to our group. Therefor one factor might be the resulting selection of subjects, which included mostly lean and athletic women. Investigating a group of males with similar small BMIs, Moller et al. [39] found similarly small IHL values. While van Herpen et al. [38] found an 17% IHL increase after a three week high-fat diet we could not observe significant changes of IHL after 6 weeks of KD in healthy subjects. The IHL values were below 3% in all and below 1% in 7 of 11 subjects. Even though the average change in body weight was >2kg, the calculated IHL changes between PRE and POST examinations were even smaller and heterogeneous across subjects. Within the accuracy achievable with the given setup, there are no significant changes in IHL between PRE and POST examinations. 

In the literature, it has been reported that a diet rich in saturated fats can increase both liver fat and insulin resistance [40] in patients. With different dietary approaches, the intrahepatic lipid content not only may be reduced but furthermore intrahepatic lipid quality may be specifically modulated [18] in non-alcoholic steatohepatitis patients. However, the clinical consequences of such interventions have to be investigated in further studies. In this study, changes in the fat deposits of the liver by a ketogenic diet in healthy humans could not be detected.

Body cell mass as a compartment of fat-free mass measured by BIA was unaffected, which represents the protein-rich and metabolically-active compartment [41]. Together with a rise in hand grip strength as a surrogate marker of total muscle mass and function, the main study concluded that the KD intervention affected neither muscle mass nor muscle function negatively.

Based on these combined results, including physical fitness of the main study, the conclusion that there was no negative impact neither on muscle mass nor on muscle function, and only a mild/slight negative impact on physical performance by the intervention does match with this more detailed MRI data. The body composition changes may be regarded as positive. However, the main study lacked the possibility for interpreting the fat mass loss regarding health implications, due to the indirect nature of the applied methods used to measure total body fat mass. Fat mass, a loose connective tissue composed mostly of adipocytes, can be subdivided into several compartments regarding its location: beneath the skin (subcutaneous fat), around internal organs (visceral fat), in bone marrow, intermuscular, and in the breast tissue. Fat mass is recognized as a hormonally active tissue that is able to produce hormones such as leptin, estrogen, resistin, and the cytokine TNF-alpha [42]. We found a significant median abdominal fat mass loss of 0.8 kg through MRI usage, whereby the major part of it was subcutaneous fat (75%) and the remained loss affected the internal/visceral fat.

Both fat compartments have different health implications, where excess visceral fat is associated with type 2 diabetes [43], insulin resistance [44], inflammatory diseases [45], and other obesity-related diseases [46]. Hence, from a clinical perspective, preferential loss of visceral fat may be metabolically advantageous [47]. Interestingly, the fat mass loss during the KD intervention led mostly to a decrease in subcutaneous fat, which is not related to many of the classic obesity-related pathologies [48]. Especially notable is the fact that a non-energy-restricted KD without any changes in physical activity resulted in losses of abdominal subcutaneous and intraabdominal fat mass.

Magnetic resonance spectroscopy results were unexpectedly of limited use. The aim to use additional information to characterize the hepatic fat composition was only partly achieved. As shown in Figure 4, MRS was able to distinguish 6 to 10 hepatic fat components. However, except for the main contribution of L13, the other components often were very low and therefore their estimation subject to increased uncertainty. In the next step, this uncertainty prevented reliable detection of any possible changes. Subjects in other studies using MRS to monitor liver fat properties mostly were obese and only the main or overall fat component was quantified instead of individual fat components [49]. Retrospectively analyzed, the volunteer cohort was biased toward athletic, nutrition-conscious, mainly women of normal weight levels, with lifestyles dependent on low liver fat values. The signal-to-noise ratio of the investigated group therefore was much lower compared to other groups measured with a very similar protocol before [50]. For reliable measurement of such low hepatic lipid levels, one would have to increase measurement time or voxel size or both. The former is limited by patient compliance and the latter by size and structure of the liver. 

## 5. Conclusions

In summary, our results demonstrated that magnetic resonance imaging provides body composition information on the abdominal fat distribution changes during a ketogenic diet. This information is complementary to anthropomorphic and laboratory measures and it is more detailed than measures from ADP and BIA. It was shown that there was no significant change in internal fat distribution as well as in intrahepatic lipid content. However, a systematic decrease in subcutaneous masses which translates into a decrease in total mass equaling to moderate body weight reduction was found. Magnetic resonance spectroscopy however was of limited use in the investigated cohort, which might require a modified MRS acquisition protocol for reliable detection of small lipid components. This might be different in more adipose subjects with higher lipid levels in the liver.

## Figures and Tables

**Table 1:** PRE and POST mass values estimated by the three modalities BIA, ADP, and MRI.

	Unit	PRE	POST	*Asymp. Significance (2-Tailed)*
Body weight	kg	75.6 (58.5–89.7)	73.1 (56.0–86.5)	0.003
BMI	kg/m^2^	26.1 (23.2–28.6)	25.2 (22.2–27.3)	0.003
**Bioelectrical Impedance Analysis (BIA)**				
Fat mass	kg	26.1 (17.7–35.2)	23.9 (16.3–31.4)	0.003
Fat-free mass	kg	47.2 (39.9–71.9)	48.9 (38.3–70.2)	0.003
**Air Displacement Plethysmography (ADP)**				
Fat mass	kg	28.7 (21.5–38.2)	27.3 (19.2–36.5)	0.075
Fat-free mass	kg	46.9 (36.7–68.2)	45.8 (36.1–67.3)	0.075
**Magnetic Resonance Imaging (MRI**)				
Abdominal mass total	kg	22.5 (16.1–28.2)	21.7 (15.2–26.9)	0.929
Abdominal fat mass total	kg	11.2 (6.5–14.6)	10.5 (6.3–13.2)	0.016
Abdominal fat-free mass total	kg	11.3 (9.0–15.8)	11.3 (8.9–15.7)	0.013
Mass subcutaneous	kg	7.1 (4.2–9.7)	6.6 (3.9–8.9)	0.004
Fat mass subcutaneous	kg	6.8 (3.7–9.5)	6.2 (3.3–8.7)	0.004
Fat-free mass subcutaneous	kg	0.3 (0.2–0.6)	0.4 (0.2–0.7)	0.004
Mass internal	kg	15.3 (11.8–21.3)	15.1 (11.3–20.4)	0.026
Fat mass internal	kg	4.4 (2.8–6.2)	4.2 (2.7–5.5)	0.799
Fat-free mass internal	kg	10.9 (8.8–15.2)	10.9 (8.3–15.0)	0.021

The mean values with minimum to maximum ranges in brackets are listed. Before statistical testing values were normalized to the total body weight. Asymptotic significance values (2-tailed) based on Wilcoxon Signed Rank test are given. Parameter with systematic differences between PRE vs. POST values are marked as *cursive*. MRI data results from restricted acquisition volume within the trunk. BIA and ADP values are estimations for the whole body. Body Weight and BMI data is given for comparison.

## Supplementary Materials

The following are available online at https://www.mdpi.com/2072-6643/12/1/244/s1, Figure S1. Scout image (right) with inscribed location of Dixon fat/water MRI (orange) and liver MR spectroscopy voxel (yellow). The light blue box is the location of the navigator acquisition volume used during the MRS acquisition. Figure S2. Changes of individual subjects BMI. X-axis labels PRE and after 6 weeks POST values. For better visualization of the changes between PRE and POST the Y-axis is limited to the range of the values. Figure S3. Bland-Altman plots for changes in fat mass (top) and fat-free mass (bottom) between baseline and follow up examination based on ADP, BIA and MRI measurements. In all except of one subject a systematic decrease of fat mass (top row) is seen in all modalities. Fat-free mass (bottom row) shows a similar decreasing development in ADP and MRI data, while BIA data displays an inconsistent development. Figure S4. Bland-Altman plots for fat (top) and fat-free mass (bottom) estimated from ADP and BIA measurements. Comparing results of both modalities show a positive bias for fat mass and a negative for fat-free mass.
