# Supplementary material for: In Vivo Fat Quantification: Monitoring Effects of a 6-Week Non-Energy-Restricted Ketogenic Diet in Healthy Adults Using MRI, ADP and BIA
Source: Nutrients. 2020 Jan 17;12(1):244. doi: 10.3390/nu12010244 (PMC7019649; doi:10.3390/nu12010244)
Supplement: Supplementary file 1 [file nutrients-12-00244-s001.pdf]

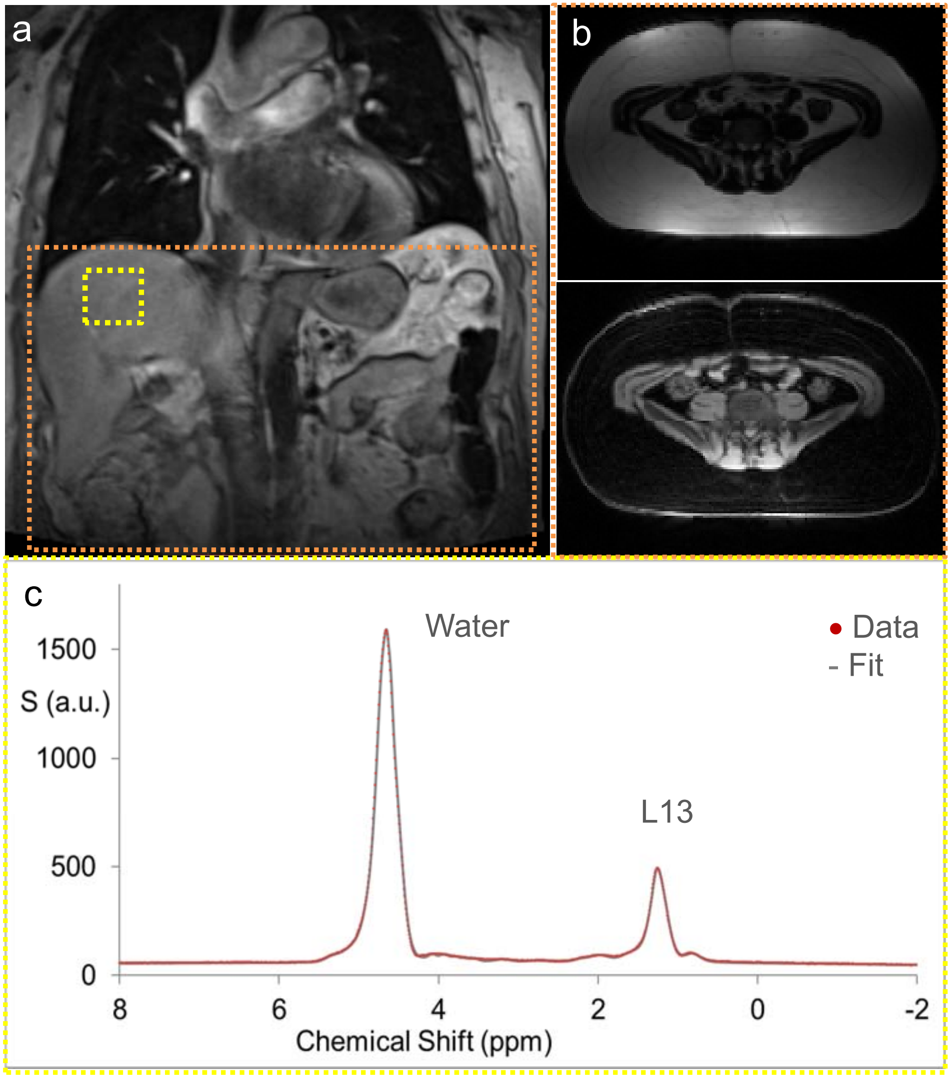

Figure S1. (a) MR localizer image with inscribed location of Dixon fat/water MRI acquisition volume (dashed orange) and liver MR spectroscopy voxel (dashed yellow).
(b) Exemplary axial fat (top) and water (bottom) MRI image of a single slice out of the Dixon fat/water MRI acquisition volume shown in (a).
(c) MR spectra without water suppression of the liver voxel shown in (a). Red dots are original data; Underlying gray line are the model fit of the data. Due to the strong water signal the only lipid signal clearly visible with this scaling is L13.

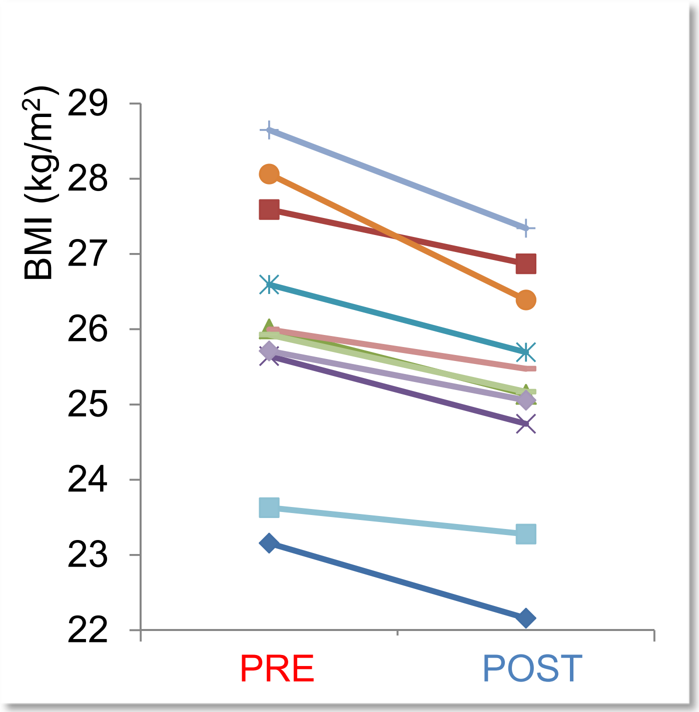

Figure S2. Changes of individual subjects BMI. X-axis labels PRE and after 6 weeks POST values. For better visualization of the changes between PRE and POST the Y-axis is limited to the range of the values.

Figure S3. Bland-Altman plots for changes in fat mass (top) and fat free mass (bottom) between baseline and follow up examination based on ADP, BIA and MRI measurements. In all except of one subject a systematic decrease of fat mass (top row) is seen in all modalities. Fat free mass (bottom row) shows a similar decreasing development in ADP and MRI data, while BIA data displays an inconsistent development.

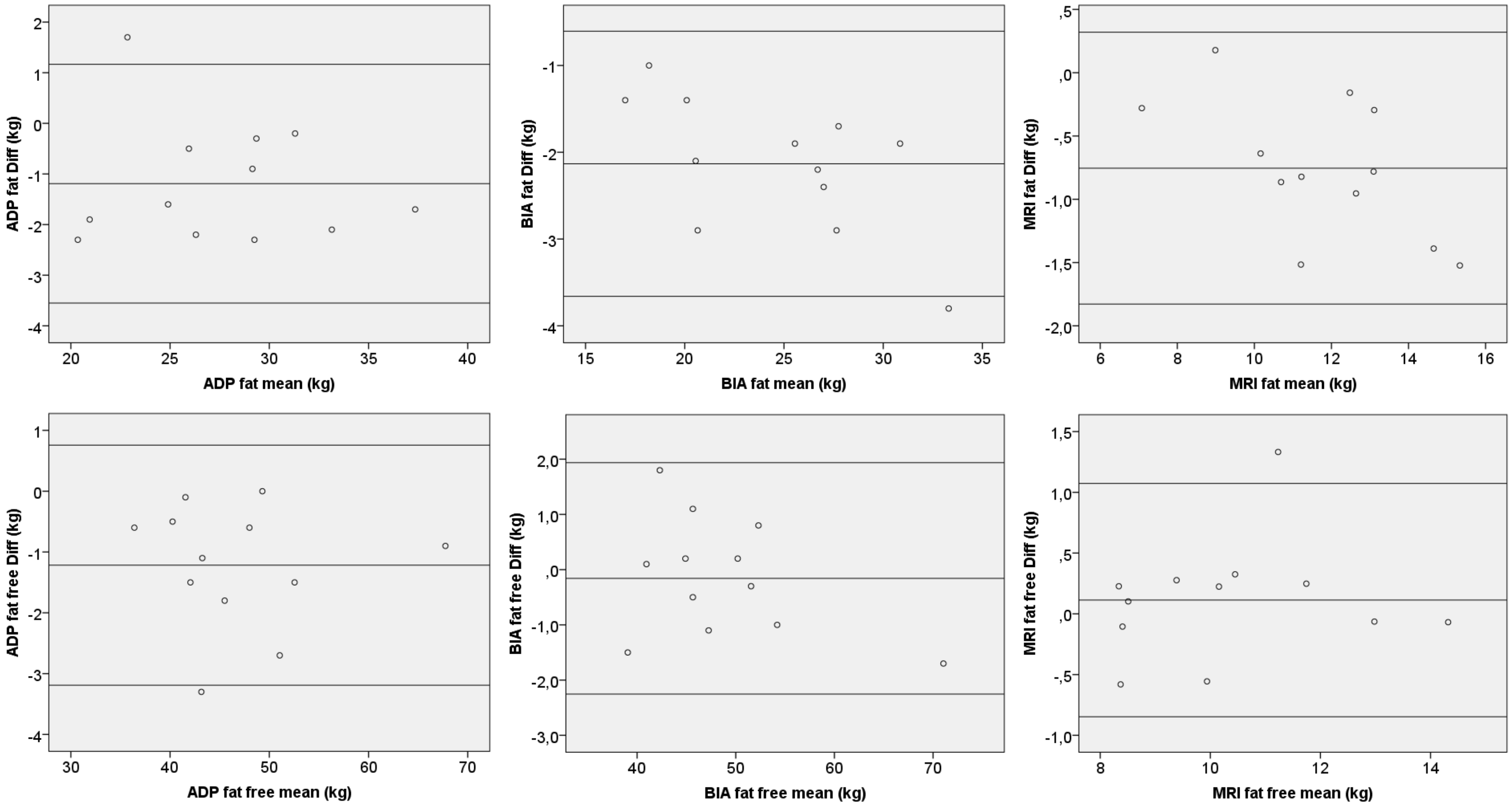

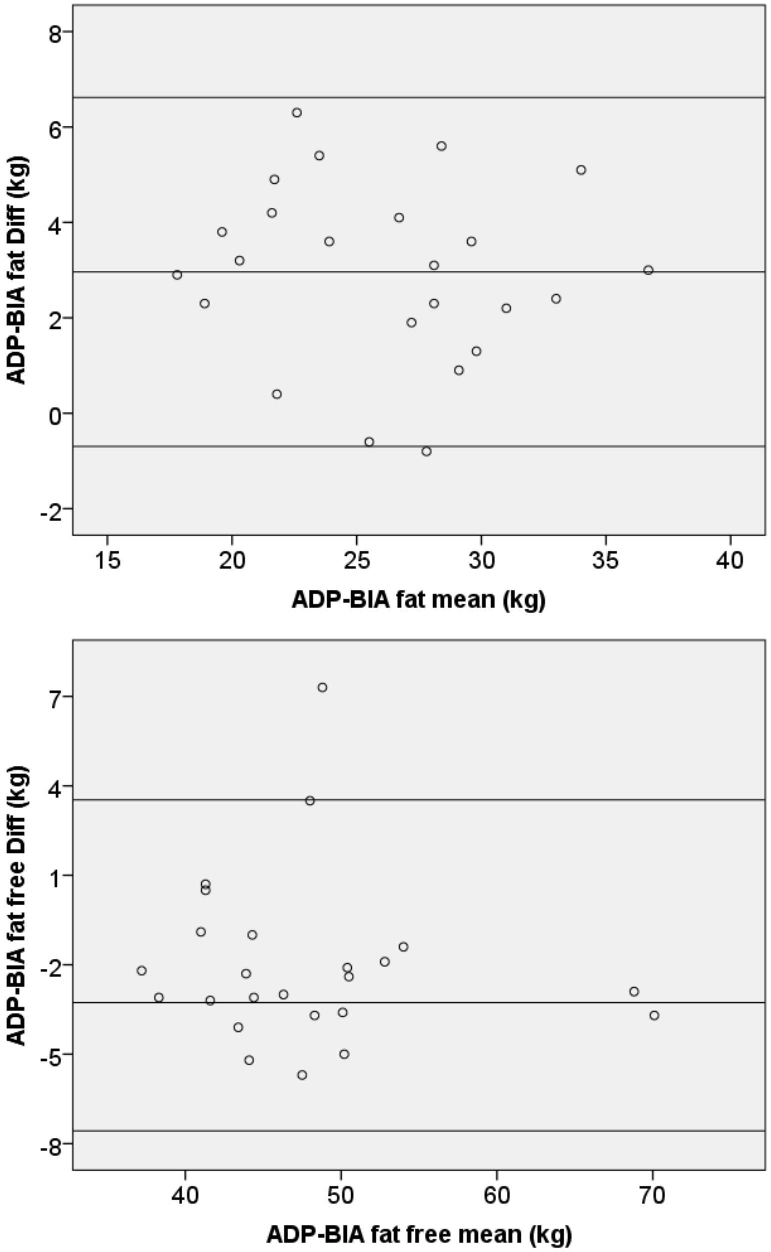

Figure S4. Bland-Altman plots for fat mass (top) and fat free mass (bottom) estimated from ADP and BIA measurements. Comparing results of both modalities show a positive bias for fat mass and a negative for fat free mass.
